# Dose–response effects of light therapy on sleepiness and circadian phase shift in shift workers: a meta-analysis and moderator analysis

**DOI:** 10.1038/s41598-021-89321-1

**Published:** 2021-06-07

**Authors:** Calvin Lam, Min-Huey Chung

**Affiliations:** 1 Department of Psychology, The University of Hong Kong, Pok Fu Lam, Hong Kong; 2 School of Nursing, College of Nursing, Taipei Medical University, Taipei, Taiwan; 3 Department of Nursing, Shuang Ho Hospital, Taipei Medical University, New Taipei City, Taiwan

**Keywords:** Therapeutics, Circadian rhythms and sleep

## Abstract

Light therapy has been considered to be effective in mitigating sleepiness and regulating circadian phase shift in shift workers. However, the effective treatment dose of light therapy remains undetermined. We performed a meta-analysis of randomized experimental studies to determine the effect of light therapy doses on sleepiness and circadian phase shift in shift workers. An article search was performed in 10 electronic databases from inception to June 2020. Two raters independently screened and extracted data and reached consensus. Twenty-one eligible studies were included. Analyses were performed using random-effects models. Light therapy exerted significantly small to medium effects on sleepiness and large treatment effects on circadian phase shift. Moderator analyses performed with subgroup and metaregression analyses revealed that medium-intensity light therapy for a shorter duration more effectively reduced sleepiness at night, whereas higher-intensity light therapy more effectively induced phase shifting, but the required treatment duration remained inconclusive. This study provides evidence regarding the effect of light therapy in reducing sleepiness and shifting circadian phase in shift workers. Exposure to medium-intensity light for a short duration at night reduced sleepiness, whereas exposure to high-intensity light improved sleep by shifting their circadian phase.

## Introduction

Shift work involves staff members working during the day, evening, or night, enabling an organization to operate longer than individual workers can work^1–3^. Quick-rotating night shifts may reduce the attentional performance of the shift workers because of the generation of a higher level of prolactin (a sleep-related hormone) in daytime; in addition, fatigue, daytime sleepiness, and insomnia have been found in shift workers^4–7^. Scheduled shift work is required in many industries, such as the nursing sector; therefore, mitigating the sleep problems of shift workers is crucial to sustain their energy and health status.

Sleepiness and circadian phase problems are two critical examples of sleep problems in shift workers. Sleepiness is a condition marked by inactivity and a tendency to fall asleep^8,9^. Reducing the sleepiness of shift workers during shift work can increase work performance and reduce accidents^4,10^. Light therapy is a nonpharmacological treatment that has been considered effective in mitigating the sleepiness of shift workers. The use of light exposure at nighttime to delay the sleep phase may increase alertness for working at night and increase daytime sleepiness^11,12^. By contrast, light exposure during daytime to advance the sleep phase may regulate and increase nighttime sleepiness on days off^13^. After light exposure, shift workers may exhibit improved alertness at work and improved sleep after work and on days off.

The circadian phase problem is a misalignment between the circadian clock and sleep–wake schedule^13^. For instance, individuals have a normal sleep–wake schedule of sleeping at nighttime and wake up in the morning; however, shift workers may work at night, sleep in the morning, and wake up in the afternoon or evening. Shifting the circadian phase to an adaptive state for shift work may reduce sleep problems caused by shift work. Individuals exposed to artificial bright light or sunlight under light therapy have exhibited a circadian phase shift^13–17^ because exposure to light may have suppressed melatonin and shifted the sleep phase^11,12^. Hence, light therapy may adjust the timing of melatonin secretion to coincide with the sleep phase.

To effectively use light therapy to enhance the sleep health of shift workers, understanding whether light therapy exerts a dose–response effect on the sleep problems of shift workers is crucial. However, this is a debated topic. The dose of light therapy is measured on the basis of light intensity (lux) and treatment duration, expressed as the total hours of light exposure^11,18,19^. A study indicated that different light intensities may not exert different treatment effects on sleep problems^20^. In addition, a meta-analysis revealed that treatment effects did not significantly differ among patients with seasonal affective disorder when treated with strong (6000–10,000 lux), medium (1700–3500 lux), and dim light (≤ 600 lux)^18^. The findings of the aforementioned studies indicate that differences in light intensity might not determine the treatment effects of light therapy. However, some recent studies have revealed that differences in light duration may exert different treatment effects on phase delay of the circadian rhythm and subjective sleepiness^11,19^. A longer duration of light exposure (1–3 h) increased the magnitude of circadian phase shift, however a higher light intensity (2000–8000 lux) did not further increase the magnitude^11^. Although a longer duration of light exposure (0.2–4 h) more effectively suppressed melatonin secretion and reduced sleepiness, the effectiveness diminished with a further increase in exposure duration^19^.

By examining randomized studies, previous meta-analyses have investigated the effects of pharmacological and nonpharmacological interventions on sleep-related outcomes in shift workers^1,2^. However, evidence regarding the effects of the dose–response and related moderators of light treatment on sleepiness and circadian phase shift in shift workers is limited. In particular, whether different intensities and durations of light exposure have different dose–response relationships with sleepiness and circadian phase shift remains unclear. The present meta-analysis examined potential variations in the dose–response effects of light therapy on sleepiness and circadian phase shift in shift workers.

## Methods

This meta-analysis adhered to the criteria of the Preferred Reporting Items for Systematic Reviews and Meta-Analyses statement^21^.

### Search strategy

Ten electronic databases, namely PubMed, CINAHL, Medline, SCOPUS, PsycInfo, Cochrane, Pubpsych, Opengrey, LILACS, and Embase, were searched for the articles for potential inclusion. The search strategy was limited to human studies and clinical trials. A specific search strategy was developed for each database on the basis of a combination of search terms ([“light therapy”, “phototherapy”, or “light”], [“shift work”, “shift”, “shift worker”, “work schedule tolerance”, or “night work”], and [“randomized clinical trial” or “random”]). The search was conducted in June 2015, and two update searches were conducted in April 2016 and June 2020. Articles from journals and conferences were selected. In addition, no restrictions on the written language (necessary translation was performed after the search) or publication date (from inception to June 2020) were applied. The current meta-analysis used the following three-step search strategy: first, papers were searched and collected from the aforementioned databases and the relevant trial registers, references, and websites of the study organizations and institutions; second, the titles and abstracts of the searched studies were verified on the basis of inclusion criteria; and finally, data were extracted from the included studies.

### Study screening

Eligibility criteria for the included studies were screened in accordance with the PICO process. Participants were individuals who were shift workers (night shift or rotating shift) or worked in simulated shifts. The interventions were those using light therapy with a single or mixed type of light. The studies made comparisons with a differentiable light intensity of ≤300 lux or no active treatment in the control group (the treatment of the control group was not similar to that of the experimental group). Randomization was ensured by including experimental studies involving randomized controlled trials or randomized crossover trials. For outcomes, the included studies had adequate data for the outcome variables of sleepiness or circadian phase shift for calculating effect sizes.

### Risk-of-bias assessment

Two raters independently assessed the risk of bias for each included study using the criteria provided in the Cochrane Handbook for Systematic Reviews of Interventions, Version 5.1.0^22^. Each criterion was qualified as being low, high, or unclear. A study was excluded if all the criteria were identified as “high risk”, which indicated that the study was not conducted in a rigorous setting. Consensus was reached through discussion.

### Data extraction

Two raters independently performed the database search and screened the titles and abstracts of the retrieved studies. The two raters assessed the eligibility of the records by reviewing the full texts of the studies. Inconsistencies were resolved through discussion. The two raters independently performed data extraction and finalized the data after reaching a consensus.

### Statistical analysis

The current meta-analysis used the pooled estimates (indicated by the pooled effect size) of treatment effects after combining all included studies. Extracted data were entered into Comprehensive Meta-Analysis, Version 3.0. Random-effects models were used that assumed the samples of included studies were drawn from different populations (shift workers) in which variation and estimated uncertainty were accounted for in underlying effects. The pooled effect size, indicated by Hedges’s g (g; 0.2–0.5 = small effect, 0.5–0.8 = medium effect, and > 0.8 = large effect), was calculated using sleepiness or circadian phase shift as the outcome in shift workers. The significance level was set to p < 0.05. The directions of the outcomes were classified as “favors light therapy” (less sleepiness or more alertness and phase delay at night or phase advance during the day) and “favors control” (no effect or opposite effects on sleepiness or phase shift). Data of each study are presented as the mean, standard deviation (SD), F test results, or t test results of the experimental group and the control group for effect size calculations. The Q test and I^2^ statistics were used to examine between-study heterogeneity, where Q > 0.05 and I^2^ ≤ 50% indicated low heterogeneity and I^2^ > 50% indicated substantial heterogeneity. Publication bias was examined using Egger’s test. Potential publication bias was adjusted using Duval’s trim-and-fill method. Sensitivity analyses were performed by removing individual studies to examine whether significance and pooled effect size were affected. Subgroup analyses with mixed-effects analysis (at least two studies were included in each subgroup) for categorical moderators and metaregression with mixed models (unrestricted maximum-likelihood) were performed to assess categorical and continuous moderators. Light therapy dose was indicated by light intensity, duration, and their combination (i.e. lux hours = lux × hours)^23^ for examining the dose–response effect of light therapy on sleepiness and circadian phase shift by using subgroup analyses.

### Ethics declarations

No human or animal subjects were directly involved in this study.

## Results

### Study characteristics

**Figure 1:**
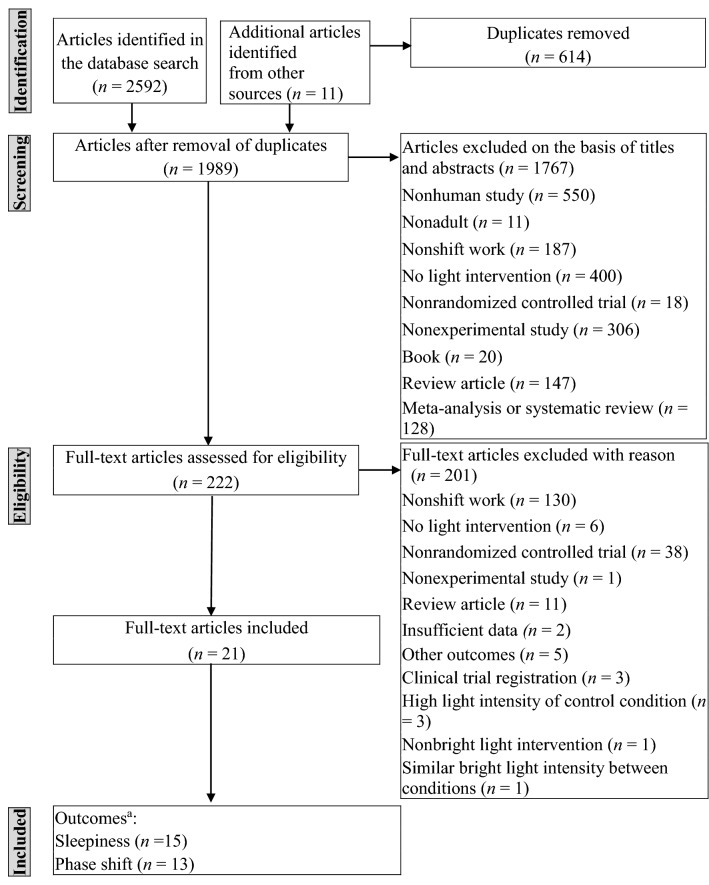
Flow diagram of study inclusion and exclusion in the meta-analysis ^a^Some studies included more than one outcome.

A flow diagram illustrating the process of study selection (inclusion and exclusion of studies) for this meta-analysis is presented in Fig. 1. A total of 2603 articles was obtained from 10 databases and references of the articles. A total of 21 studies meeting the eligibility criteria were included^9,13–17,24–38^. The characteristics of the participants in the included studies are listed in Table 1. The total number of participants was 656, with a mean age of 31.51 years (SD = 5.87). The participants were shift workers or participants who performed simulated shift work during experiments. The intervention characteristics of the included studies are presented in Table 2. The studies used bright light, white light, nocturnal light, and sunlight as intervention treatments and light intensities between 430 and 10,000 lux. Furthermore, the studies used a single type of light or a combination of dim light, room light, white light, sunlight, placebo capsules, and no active treatment as the control treatment. The range of light intensity for active control treatment was 1–300 lux. The treatment duration was identical for both the experimental and control groups in each study. The total duration of exposure was between 0.5 and 42 h. Some eligible studies included both outcomes, namely sleepiness and circadian phase shift. Fifteen studies recorded the outcome of sleepiness. These studies recorded the outcome by using the following subjective scales: the Karolinska Sleepiness Scale (KSS) with 9-point scales from “very alert” to “very sleepy, fighting sleep, an effort to stay awake;” the Stanford Sleepiness Scale (SSS) with 7-point scales from “feeling active and vital, alert, wide awake” to “lost struggle to remain awake;” and a visual analog scale (VAS) using 10-cm or 100-cm scales with adjectives from “sleepy” to “alert”. Thirteen studies recorded the outcome of circadian phase shift, which was measured by determining melatonin secretion (from a salivary or urine sample) by using a radioimmunoassay or enzyme-linked immunosorbent assay or by examining core temperature. In the current study, negative values of effect sizes represented “favors control condition,” whereas positive values represented “favors light therapy.” For publication bias, after Egger’s test was conducted, significant publication bias was found for sleepiness (p < 0.001) but not for phase shift (p = 0.053). No potential missing study was identified for the effect of light therapy on outcomes in the funnel plot by using Duval’s trim-and-fill method.

**Table 1:** Study characteristics.

Characteristics	Mean	SD
**Participants (n = 656)**		
Age (years)^a^	31.51	5.87

	*n*	%
**Sex**^**a**^		
Female	273	42.66
Male	367	57.34
**Sample size**^**b**^		
Experimental group	438	NA
Control group	435	NA
**Study (n = 21)**		
Type of shift work		
Rotating shift	7	33.33
Night shift	14	66.67
Simulated shift		
Yes	12	57.14
No	9	42.86
Treatment period		
Day only or mixed time	10	47.62
Night only	11	52.38
Research design		
Randomized controlled trial	9	42.86
Randomized crossover trial	12	57.14
Use of intention-to-treat analysis		
Intention-to-treat	10	47.62
Per protocol	11	52.38

*SD *standard deviation, *NA *not applicable.

^a^Missing value in (number of study): mean age (3), SD age (4), sex (1).

^b^Sample size: participants were included in both intervention and control groups in randomized crossover trials.

**Table 2:** Intervention characteristics of the included studies.

Study	Intervention treatment	Comparison treatment	Sample size (*n*)	Treatment period	Shift work period	Measure period
Babkoff 2002	BL and caffeine^a^	DL and caffeine	EG: 6 CG: 6	2330–0230^c^	1730–0830^f,h^	0130–0430
Bjorvatn 2007	BL^b^	Placebo capsules	EG: 17 CG: 17	0000–0500 and 1200–1430^c^	0630–1830 and 1830–0630^g^	Every 2 h from 2000 and so on
Bjorvatn 2020	BL	DL	EG: 35 CG: 35	0200–0300, 0300–0400, 0400–0500	2200–0600	every 2 h at 2200–0600
Boivin 2012	Intermittent BL^a^	No	EG: 8 CG: 9	6 h from 2200, 2230, 2300, or 2330^c^	8/8.5 h between 2200–0800^f^	Every 2 h (phase shift) or every 30 min (sleepiness) at 0600–2200
Comtet 2019	BL	DL	EG: 18 CG: 18	0500–0530	2000–0800	0300, 0500, 0700, 0800
Dawson 1991	BL and DL^a^	DL	EG: 6 CG: 7	2400–0400^c^	2300–0700^f,h^	0100, 0300, 0500, and 0700
Dawson 1995	BL^a^	DL	EG: 8 CG: 8	2400–0400^c^	2300–0700^f,h^	At night
Griepentrog 2018	BL	SL	EG: 26 CG: 17	1900–0700	1900–0700	0500, 0700
Horowitz 2001	BL and fixed or free sleep	RL and fixed or free sleep	EG: 25 CG: 27	2300–0700	2300–0700, 0700–1100	2300–0700
Karchani 2011	BL^b^	RL	EG: 45 CG: 45	2200, 2400, 0200 and 0400^c^	0600–1400, 1400–2200, and 2200–0600^ g^	2300, 0100, 0300, and 0500
Kretschmer 2013	BL and RL^a^	RL	EG: 16 CG: 16	2200–0200, 2300–0300, 0000–0400^c^	2200–0400^f,h^	Every 2 h/day

Study	Illuminance (lux)	Treatment days	Tool	Outcome		
Babkoff 2002	E: 3000 C: 20–50	1	VAS and RIA	Sleepiness and phase shift		
Bjorvatn 2007	E: 10,000 C: 0	8	KSS	Sleepiness		
Bjorvatn 2020	E: 10,000 C: 100	3	KSS	Sleepiness		
Boivin 2012	NA	7	VAS and RIA	Sleepiness and phase shift		
Comtet 2019	E: 10,000 C: 8	1	KSS	Sleepiness		
Dawson 1991	E: BL 6000 and DL 150–200 C: 150–200	3	Core temperature	Phase shift		
Dawson 1995	E: 4000–7000 C: 50	3	RIA	Phase shift		
Griepentrog 2018	E: 1500–2000 C: 300	1	SSS and ELISA	Sleepiness and phase shift		
Horowitz 2001	E: 2500 C: 150	3	VAS and RIA	Sleepiness and phase shift		
Karchani 2011	E: 2500–3000 C: 300	2	SSS	Sleepiness		
Kretschmer 2013	E: BL 3000 and RL 300 C: 300	3	SSS	Sleepiness		

Study	Intervention treatment	Comparison treatment	Sample size (n)	Treatment period	Shift work period	Measure period
Lee 2006	BL	DL	EG: 11 CG: 12	0045–0500	2300–0700	Every 30 min at 1530–1200; 0000–1800 in last day
Lee 2020	BL	DL	EG: 12 CG: 12	0100–0600	0100–0600	Every hour at 2100–0600
Lowden 2004	BL	RL	EG: 18 CG: 18	1515–2145, 2145–0630, or 2400–0630	1515–2145, 2145–0630, and 2400–0630	Every 2 h at 0000–0600, 0600–1400
Nagashima 2017	BL	DL	EG: 12 CG: 12	1000–1600	0000–0800	Every hour at 1800–0000
Sadeghniiat-Haghighi 2011	BL	RL	EG: 47 CG: 47	0030–0050, 0230–0250	0600–1800, 1800–0600^g^	2200, 2400, 0200, 0400
Smith 2009	Intermittent BL pulse and RL	RL	EG: 9 CG: 10	0045–0400	2300–0700	Every 30 min/day
Sunde 2020	BL	SL	EG: 36 CG: 36	2300–0500	2300–0700	2330, 0100, 0230, 0400, 0530
Tanaka 2011	BL	No	EG: 61 CG: 61	0730–0800	0800–1700, and 1630–0830	1000, 1400
Thorne 2010	WL and wearing sunglasses	No	EG: 10 CG: 10	1300–1400	1800–0600, or 1900–0700	Every 4 h/day
Yoon 2002	BL	RL	EG: 12 CG: 12	0100–0500 and 0830–0930	2200–0800	4 times at 2400–0600

Study	Illuminance (lux)	Treatment days	Tool	Outcome		
Lee 2006	E: 3500 C: < 50	2	RIA	Phase shift		
Lee 2020	E: 430 C: < 1	1	KSS and RIA	Sleepiness and phase shift		
Lowden 2004	E: 2500 C: 300	15	KSS and RIA	Sleepiness and phase shift		
Nagashima 2017	E: > 3000 C: < 50	1	RIA	Phase shift		
Sadeghniiat-Haghighi 2011	E: 2500 C: 300	1	SSS	Sleepiness		
Smith 2009	E: 4100 C: < 50	8	RIA	Phase shift		
Sunde 2020	E: 900 C: 90	3	KSS and ELISA	Sleepiness and phase shift		
Tanaka 2011	E: 5444–8826 C: 0	30	KSS	Sleepiness		
Thorne 2010	E: 3000 C: 0	4	RIA	Phase shift		
Yoon 2002	E: NL 4000–6000 and SL 10,000 C: RL < 200 and SL 10,000	3	VAS	Sleepiness		

*BL *bright light, *DL *dim light, *WL *white light, *NL *nocturnal light, *SL *sunlight, *RL *room light, *E *experimental group, *C *control group, *No* no treatment, *NA *not available, *ELISA *enzyme-linked immunosorbent assay, *KSS *Karolinska Sleepiness Scale, *RIA *Radioimmunoassay, *SSS *Standford Sleepiness Scale, *VAS *Visual Analog Scale.

### Risk-of-bias assessment

The risk-of-bias assessment of the included studies is shown in Table 3. None of the studies met all the criteria. Most of the studies (all k = 19) had an unclear or high risk in terms of sequence generation, allocation concealment, blinding of participants and personnel, and blinding of outcome assessors. Subgroup analysis was not performed for these criteria because a substantial risk of bias was already demonstrated in these assessments. Five studies had a high risk of incomplete outcome data, and one study had a high risk of selective outcome reporting. The remaining studies had a low level of risk for the two criteria. Thus, subgroup analysis was performed for only incomplete outcome data for both outcomes: sleepiness (Table 5c), low risk (g = 0.474, 95% confidence interval [CI] = 0.089–0.290, p < 0.001) and high risk (g = 0.190, 95% CI = 0.339–0.609, p < 0.001); phase shift (Table 6c), low risk (g = 1.150, 95% CI = 0.718–1.583, p < 0.001) and high risk (g = 0.857, 95% CI = 0.223–1.491, p = 0.008). Overall, these results indicate that the risk of selective outcome reporting was low; however, the other five criteria of bias substantially influenced the effects of the outcomes.

**Table 3:** Risk-of-Bias assessment of included studies.

Study	Random sequence generation	Allocation concealment	Blinding of participants and personnel	Blinding of outcome assessment	Incomplete outcome data	Selective reporting
Babkoff 2002	U	U	H	L	H	L
Bjorvatn 2007	U	U	U	U	H	L
Bjorvatn 2020	U	L	L	U	H	L
Boivin 2012	U	U	U	U	L	H
Comtet 2019	L	U	U	U	L	L
Dawson 1991	U	U	U	U	L	L
Dawson 1995	U	U	L	U	L	L
Griepentrog 2018	H	U	U	U	L	L
Horowitz 2001	U	U	U	U	L	L
Karchani 2011	U	U	U	U	L	L
Kretschmer 2013	U	U	U	U	L	L
Lee 2006	U	U	U	U	L	L
Lee 2020	U	U	U	U	L	L
Lowden 2004	U	U	H	U	L	L
Nagashima 2017	U	U	U	U	L	L
Sadeghniiat-Haghighi 2011	U	U	U	U	L	L
Smith 2009	U	U	U	L	L	L
Sunde 2020	U	U	U	U	H	L
Tanaka 2011	L	L	H	U	L	L
Thorne 2010	U	U	H	U	H	L
Yoon 2002	U	U	U	U	L	L

*L *low risk, *H *high risk, *U *unclear risk.

### Treatment effects

**Figure 2:**
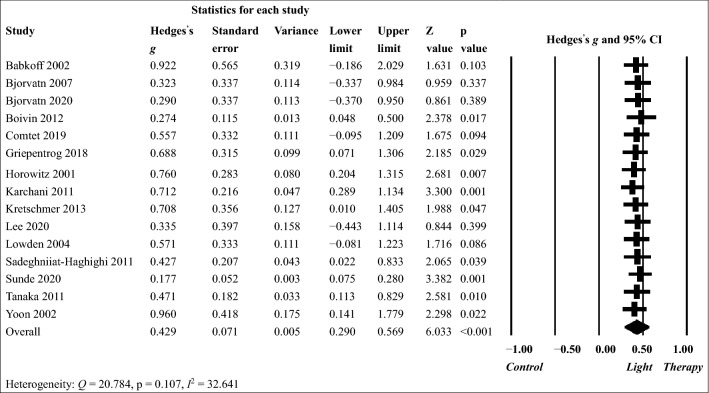
Treatment effect of light therapy on sleepiness in shift workers analyzed using a random-effects model.

**Figure 3:**
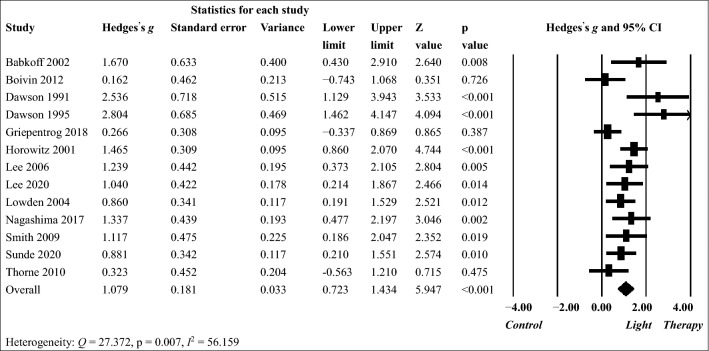
Treatment effect of light therapy on phase shift in shift workers analyzed using a random-effects model.

The results of random-effects models used for examining the effects of light therapy on sleepiness and circadian phase shift in shift workers are presented in Figs. 2 and 3, respectively. For sleepiness, light therapy had a small to medium pooled effect size (g = 0.429, 95% CI 0.290–0.569, p < 0.001) in the 15 included studies. The between-study heterogeneity was low (Q = 20.784, p = 0.107, I^2^ = 32.641). The effects of light therapy on reducing sleepiness were significant in nine studies but not in other six studies. Sensitivity analysis showed that the pooled effect size remained significant after each study was removed  (g = 0.381–0.481, all p < 0.001). For phase shift, the pooled effect size was large (g = 1.079, 95% CI 0.723–1.434, p < 0.001) in the 13 included studies. The between-study heterogeneity was substantial (Q = 27.372, p = 0.007, I^2^ = 56.159). Significantly positive effects of light therapy were observed in 10 studies but not in the other 3 studies. Similarly, sensitivity analysis showed that the pooled effect size remained similar and significant after each study was removed (g = 0.980 to 1.161, all p < 0.001).

### Moderator analyses

The moderators are listed in Table 4. The results of subgroup and metaregression analyses are presented in Tables 5 a–c, 6 a–c and 7, respectively. Overall, the subgroup analysis performed using mixed-effects models demonstrated low heterogeneity (I^2^ = 0–30.014). The metaregression analysis performed using mixed models exhibited low heterogeneity for the models of sleepiness (I^2^ = 0–36.55) and the significant moderators of phase shift (I^2^ = 29.04–39.68) but substantial heterogeneity for the nonsignificant moderators of phase shift (I^2^ = 59.67–62.44).

**Table 4:** Moderators used in subgroup and metaregression analyses.

Study	Age (mean or range)	Female (n)	Sample size (n)	Shift work	Simulated shift	Research design	Data handling	Timing of treatment	Treatment day	Measure period
Babkoff 2002	24.6	5	12	Night	Simulated	RCT	PP or unknown	Night time	Same day	Night time
Bjorvatn 2007	42	1	17	Rotating	No	Crossover	PP or unknown	Day or mixed	Different day	Night time
Bjorvatn 2020	35.4	28	35	Night	No	Crossover	PP or unknown	Night time	Different day	Day or mixed
Boivin 2012	30.06	8	17	Rotating	No	RCT	ITT	Day or mixed	Different day	Day or mixed
Comtet 2019	24.78	5	18	Night	Simulated	Crossover	PP or unknown	Day or mixed	Same day	Day or mixed
Dawson 1991	21.2	6	13	Night	Simulated	RCT	ITT	Night time	Different day	Night time
Dawson 1995	23.6	NA	16	Night	Simulated	RCT	ITT	Night time	Different day	Night time
Griepentrog 2018	26–32	22	43	Night	No	Crossover	ITT	Day or mixed	Same day	Night time
Horowitz 2001	26.99	27	54	Rotating	Simulated	RCT	PP or unknown	Day or mixed	Different day	Day or mixed
Karchani 2011	30.415	0	90	Rotating	No	Crossover	ITT	Night time	Same day	Night time
Kretschmer 2013	58.5	50	32	Night	Simulated	RCT	ITT	Night time	Different day	Day or mixed
Lee 2006	24.5	12	23	Night	Simulated	RCT	PP or unknown	Night time	Different day	Day or mixed
Lee 2020	20.65	0	24	Night	Simulated	Crossover	PP or unknown	Day or mixed	Same day	Night time
Lowden 2004	36.2	1	18	Rotating	No	Crossover	ITT	Day or mixed	Different day	Day or mixed
Nagashima 2017	24.8	0	12	Night	Simulated	Crossover	PP or unknown	Day or mixed	Same day	Night time
Sadeghniiat-Haghighi 2011	33	0	94	Rotating	No	Crossover	PP or unknown	Night time	Same day	Night time
Smith 2009	25.79	11	19	Night	Simulated	RCT	ITT	Night time	Different day	Day or mixed
Sunde 2020	19–30	24	36	Night	Simulated	Crossover	PP or unknown	Night time	Different day	Night time
Tanaka 2011	29.7	61	61	Rotating	No	Crossover	ITT	Day or mixed	Different day	Day or mixed
Thorne 2010	46.5	0	10	Night	No	Crossover	PP or unknown	Day or mixed	Different day	Night time
Yoon 2002	21–24	12	12	Night	No	Crossover	ITT	Day or mixed	Different day	Night time

Study	Control intervention	Treatment light	Light intensity of EG (lux)	Treatment duration (daily hour)	Treatment duration (total hour)	Daily treatment session (n)	Lux-hours (per 1000)			
Babkoff 2002	Dim light	Mixed	3000	1	1	1	3.00			
Bjorvatn 2007	No	Single	10,000	0.5	4	1	40.00			
Bjorvatn 2020	Dim Red light	Single	10,000	0.5	1.5	1	15.00			
Boivin 2012	No	Single	NA	6	42	1	NA			
Comtet 2019	Dim light	Single	10,000	0.5	0.5	1	5.00			
Dawson 1991	Dim light	Single	6175	8	24	1	148.20			
Dawson 1995	Dim light	Single	5500	4	12	1	66.00			
Griepentrog 2018	Standard light	Mixed	1750	10	10	1	17.50			
Horowitz 2001	Room light	Single	2500	6	18	1	45.00			
Karchani 2011	Room light	Single	2750	1	1	4	2.75			
Kretschmer 2013	Room light	Mixed	3300	2	6	1	19.80			
Lee 2006	Dim light	Single	3500	1.25	2.5	5	8.75			
Lee 2020	Dim light	Single	430	4.17	4.17	5	1.79			
Lowden 2004	Room light	Single	2500	NA	NA	NA	NA			
Nagashima 2017	Dim light	Single	3000	6	6	1	18.00			
Sadeghniiat-Haghighi 2011	Room light	Single	2500	0.67	0.67	2	1.68			
Smith 2009	Room light	Mixed	4100	1	8	4	32.80			
Sunde 2020	Standard light	Single	900	6	18	1	16.20			
Tanaka 2011	No	Single	7135	0.17	5	1	35.68			
Thorne 2010	No	Single	3000	1	4	1	12.00			
Yoon 2002	Room light	Mixed	15,000	5	15	1	225.00			

*RCT *randomized controlled trial, *Crossover *randomized crossover trial, *ITT *intention-to-treat, *PP *per protocol.

**Table 5:** Moderators of light treatment effect on sleepiness by using subgroup analyses with mixed-effects models.

(a)						
Subgroup	k	Hedges’s g	95% CI		p (Z)^a^	I^2^
			Lower	Upper		
**Intensity of light treatment (lux)**						
Group 1						
< 1000	2	0.180	0.078	0.282	< 0.001	0
1000–5000	7	0.632	0.423	0.842	< 0.001	0
> 5000	5	0.482	0.234	0.730	< 0.001	0
Group 2						
< 2000	3	0.194	0.093	0.294	< 0.001	0
2000–5000	6	0.625	0.402	0.848	< 0.001	0
> 5000	5	0.482	0.234	0.730	< 0.001	0
Group 3						
< 3000	6	0.240	0.146	0.334	< 0.001	0
3000–5000	3	0.764	0.359	1.168	< 0.001	0
> 5000	5	0.482	0.234	0.730	< 0.001	0
Treatment duration (daily hr)						
≤ 1	7	0.504	0.312	0.695	< 0.001	0
> 1	7	0.400	0.191	0.608	< 0.001	0
Treatment duration (total hr)						
≤ 10	10	0.532	0.361	0.703	< 0.001	0
> 10	4	0.309	0.095	0.523	0.005	27.071
Number of daily treatment sessions (n)						
1	11	0.400	0.240	0.560	< 0.001	0
> 1	3	0.535	0.261	0.809	< 0.001	0

(b)						
Subgroup	k	Hedges’s g	95% CI		p (Z)^a^	I^2^
			Lower	Upper		
**Timing of treatment**						
Night time	6	0.427	0.170	0.683	0.001	0
Day time or mixed	9	0.431	0.279	0.583	< 0.001	0
**Treatment day**						
Same day	6	0.575	0.347	0.804	< 0.001	0
Different day	9	0.363	0.203	0.522	< 0.001	0
**Type of treatment light**						
Single type of light	11	0.361	0.228	0.494	< 0.001	0
Mixed type of light	4	0.779	0.401	1.158	< 0.001	0
**Control treatment**						
Active treatment	12	0.511	0.312	0.710	< 0.001	0
No treatment	3	0.330	0.146	0.513	< 0.001	0
**Shift work**						
Night shift	8	0.439	0.195	0.684	< 0.001	0
Rotating shift	7	0.430	0.284	0.576	< 0.001	0
**Simulated shift**						
Yes	6	0.454	0.162	0.746	0.002	0
No	9	0.431	0.290	0.573	< 0.001	0
**Timing of measure**						
Night time	8	0.463	0.225	0.702	< 0.001	0
Day time or mixed	7	0.410	0.252	0.569	< 0.001	0
**Research design**						
Randomized controlled trial	4	0.502	0.185	0.820	0.002	0
Randomized crossover trial	11	0.426	0.257	0.595	< 0.001	0
**Data handling**						
Intention-to-treat	7	0.480	0.310	0.649	< 0.001	0
Per protocol or unknown	8	0.305	0.149	0.461	< 0.001	0

(c)						
Subgroup	k	Hedges’s g	95% CI		p (Z)^a^	I^2^
			Lower	Upper		
**Subjective scale**						
SSS	4	0.602	0.354	0.849	< 0.001	0
VAS	4	0.576	0.190	0.963	0.003	0
KSS	7	0.221	0.127	0.315	< 0.001	0
**Risk of bias**						
Incomplete outcome data						
Low risk	11	0.474	0.339	0.609	< 0.001	0
High risk	4	0.190	0.089	0.290	< 0.001	0

^a^P value for the Z test of the moderator effect.

**Table 6:** Moderators of light treatment effect on phase shift by using subgroup analyses with mixed-effects models.

(a)						
Subgroup	k	Hedges’s g	95% CI		p (Z)^a^	I^2^
			Lower	Upper		
**Intensity of light treatment (lux)**						
Group 1						
< 1000	2	0.944	0.423	1.465	< 0.001	0
1000–5000	8	0.983	0.611	1.356	< 0.001	0
> 5000	2	2.676	1.705	3.648	< 0.001	0
Group 2						
< 2000	3	0.677	0.199	1.154	0.005	0
2000–5000	7	1.134	0.829	1.440	< 0.001	0
> 5000	2	2.676	1.705	3.648	< 0.001	0
Group 3						
< 3000	4	0.710	0.362	1.058	< 0.001	0
3000–5000	6	1.204	0.853	1.555	< 0.001	0
> 5000	2	2.676	1.705	3.648	< 0.001	0
Treatment duration (daily hr)						
≤ 1	3	0.952	0.206	1.698	0.012	0.743
> 1	9	1.166	0.688	1.644	< 0.001	17.873
Treatment duration (total hr)						
≤ 10	7	1.182	0.865	1.499	< 0.001	0
> 10	5	1.166	0.282	2.050	0.010	30.014
Number of daily treatment sessions (n)						
1	9	1.137	0.607	1.667	< 0.001	18.492
> 1	3	1.130	0.627	1.633	< 0.001	0

(b)						
Subgroup	k	Hedges’s g	95% CI		p (Z)^a^	I^2^
			Lower	Upper		
**Timing of treatment**						
Night time	6	1.528	0.950	2.107	< 0.001	7.083
Day time or mixed	7	0.798	0.390	1.206	< 0.001	0
**Treatment day**						
Same day	4	0.967	0.334	1.599	0.003	0
Different day	9	1.137	0.688	1.586	< 0.001	22.871
**Type of treatment light**						
Single type of light	10	1.141	0.741	1.541	< 0.001	21.004
Mixed type of light	3	0.889	0.062	1.717	0.035	0
**Control treatment**						
Active treatment	11	1.216	0.848	1.584	< 0.001	8.3649
No treatment	2	0.245	− 0.389	0.878	0.449	0
**Shift work**						
Night shift	10	1.164	0.720	1.607	< 0.001	11.668
Rotating shift	3	0.892	0.193	1.592	0.012	5.7388
**Simulated shift**						
Yes	9	1.379	1.041	1.718	< 0.001	7.0895
No	4	0.436	0.071	0.802	0.019	0
**Timing of measure**						
Night time	8	1.194	0.635	1.753	< 0.001	16.256
Day time or mixed	5	1.016	0.592	1.441	< 0.001	0.110
**Research design**						
Randomized controlled trial	7	1.447	0.869	2.025	< 0.001	14.171
Randomized crossover trial	6	0.748	0.423	1.072	< 0.001	0
**Data handling**						
Intention-to-treat	6	1.128	0.398	1.859	0.002	26.446
Per protocol or unknown	7	1.127	0.827	1.429	< 0.001	0

(c)						
Subgroup	k	Hedges’s g	95% CI		p (Z)^a^	I^2^
			Lower	Upper		
**Risk of bias**						
Incomplete outcome data						
Low risk	10	1.150	0.718	1.583	< 0.001	15.024
High risk	3	0.857	0.223	1.491	0.008	9.249

^a^P value for the Z test of the moderator effect.

**Table 7:** Continuous moderators of light treatment effect on sleepiness and phase shift by using metaregression with mixed models.

Moderator	k	β	95% CI		p (Z)^a^	I^2^
			Lower	Upper		
**Outcome—sleepiness**						
Lux-hours (per 1000)	13	0.002	− 0.002	0.006	0.282	36.55
Light intensity (lux per 1000)	13	0.018	− 0.025	0.061	0.404	18.55
Treatment duration (daily hr)	14	− 0.019	− 0.069	0.031	0.453	18.81
Treatment duration (total hr)	14	− 0.007	− 0.017	0.004	0.192	29.12
Age (mean)	12	0.003	− 0.019	0.024	0.805	0
Female (%)	15	− 0.003	− 0.389	0.382	0.986	33.83
Sample size (n)	15	0.002	− 0.003	0.008	0.457	28.86
**Outcome—phase shift**						
Lux-hours (per 1000)	11	0.014	0.004	0.024	0.008	35.51
Light intensity (lux per 1000)	11	0.286	0.061	0.512	0.013	39.68
Treatment duration (daily hr)	12	− 0.033	− 0.173	0.108	0.649	60.54*
Treatment duration (total hr)	12	− 0.010	− 0.047	0.027	0.596	62.44*
Age (mean)	11	− 0.052	− 0.092	− 0.013	0.009	29.04
Female (%)	12	0.154	− 1.162	1.470	0.819	50.48*
Sample size (n)	13	− 0.007	− 0.034	0.021	0.627	59.67*

*Significant at p < 0.05.

^a^P value for the Z test of the moderator effect.

### Moderator analyses of dose–response effects

The dose–response effect of light therapy on sleepiness was significantly associated with different subgroups of light intensity, treatment duration, and number of daily treatment sessions (Table 5a). Higher to lower effect sizes were associated with medium, high, and low light intensity when light intensity was grouped as follows: (1) 1000–5000 lux (g = 0.632, 95% CI = 0.423–0.842, p < 0.001), > 5000 lux (g = 0.482, 95% CI = 0.234–0.730, p < 0.001), and < 1000 lux (g = 0.180, 95% CI = 0.078–0.282, p < 0.001); (2) 2000–5000 lux (g = 0.625, 95% CI = 0.402–0.848, p < 0.001), > 5000 lux (g = 0.482, 95% CI = 0.234–0.730, p < 0.001), and < 2000 lux (g = 0.194, 95% CI = 0.093–0.294, p < 0.001); and (3) 3000–5000 lux (g = 0.764, 95% CI = 0.359–1.168, p < 0.001), > 5000 lux (g = 0.482, 95% CI = 0.234–0.730, p < 0.001), and < 3000 lux (g = 0.240, 95% CI = 0.146–0.334, p < 0.001). A higher effect size was associated with a shorter treatment duration as follows: (1) daily treatment duration ≤ 1 h (g = 0.504, 95% CI = 0.312–0.695, p < 0.001), and daily treatment duration > 1 h (g = 0.400, 95% CI = 0.191–0.608, p < 0.001) and (2) treatment duration in total ≤ 10 h (g = 0.532, 95% CI = 0.361–0.703, p < 0.001) and treatment duration in total > 10 h (g = 0.309, 95% CI = 0.095–0.523, p = 0.005). A relatively higher effect size was associated with a higher number of daily treatment session: session > 1 (g = 0.535, 95% CI = 0.261–0.809, p < 0.001) and session = 1 (g = 0.400, 95% CI = 0.240–0.560, p < 0.001). However, continuous moderators of lux hours, light intensity, treatment duration, mean age, percentage of female participants, and sample size on sleepiness were not found to be significant in metaregression analyses (all p > 0.05, Table 7). These results suggest nonlinear effects and potential saturation of light intensity (medium lux) and treatment duration (lower duration) for achieving a more favorable effect of light therapy on sleepiness, whereas a higher number of treatment sessions was more effective.

The dose–response effect of light therapy on phase shift was significantly associated with different subgroups of light intensity, treatment duration, and number of daily treatment sessions (Table 6a). Higher to lower effect sizes were associated with high, medium, and low light intensity when light intensity was grouped as follows: (1) > 5000 lux (g = 2.676, 95% CI = 1.705–3.648, p < 0.001), 1000–5000 lux (g = 0.983, 95% CI = 0.611–1.356, p < 0.001), and < 1000 lux (g = 0.944, 95% CI = 0.423–1.465, p < 0.001); (2) > 5000 lux (g = 2.676, 95% CI = 1.705–3.648, p < 0.001), 2000–5000 lux (g = 1.134, 95% CI = 0.829–1.440, p < 0.001), and < 2000 lux (g = 0.677, 95% CI = 0.199–1.154, p = 0.005); and (3) > 5000 lux (g = 2.676, 95% CI = 1.705–3.648, p < 0.001), 3000–5000 lux (g = 1.204, 95% CI = 0.853–1.555, p < 0.001), and < 3000 lux (g = 0.710, 95% CI = 0.362–1.058, p < 0.001). A higher effect size was associated with a longer daily treatment duration: > 1 h (g = 1.166, 95% CI = 0.688–1.644, p < 0.001) and ≤ 1 h (g = 0.952, 95% CI = 0.206–1.698, p = 0.012). However, a slightly higher effect size was associated with a shorter total treatment duration: ≤ 10 h (g = 1.182, 95% CI = 0.865–1.499, p < 0.001), and > 10 h (g = 1.166, 95% CI = 0.282–2.050, p = 0.010). A slightly higher effect size was associated with a fewer number of daily treatment session: session = 1 (g = 1.137, 95% CI = 0.607–1.667, p < 0.001) and session > 1 (g = 1.130, 95% CI = 0.627–1.633, p < 0.001). Significant effects of continuous moderators on phase shift were observed in metaregression analyses (Table 7): lux hours (coefficient = 0.014, 95% CI = 0.004–0.024, p = 0.008), light intensity (coefficient = 0.286, 95% CI = 0.061–0.512, p = 0.013), and mean age (coefficient = − 0.052, 95% CI = − 0.092 to − 0.013, p = 0.009); however, the effects were not significant for the percentage of female participants and sample size (all p > 0.05). These results suggest a positive association between higher light intensity and better light therapy effect on phase shift, and fewer treatment sessions were slightly more beneficial. However, the effect of treatment duration on phase shift was inconclusive. The effect of lux hours was lower than that of light intensity. These results indicat that the treatment duration did not exert a positive effect on phase shift. Moreover, younger participants experienced better treatment effects on phase shift than did older participants.

### Moderator analyses of treatment settings and research design

For sleepiness, the treatment effect was significant in the following subgroups (Table 5b): (1) timing of treatment, nighttime (g = 0.427, 95% CI = 0.170–0.683, p = 0.001) and daytime or mixed (g = 0.431, 95% CI = 0.279–0.583, p < 0.001); (2) treatment day, same day (g = 0.575, 95% CI = 0.347–0.804, p < 0.001) and different day (g = 0.363, 95% CI = 0.203–0.522, p < 0.001); (3) type of treatment light, single type (g = 0.361, 95% CI = 0.228–0.494, p < 0.001) and mixed type (g = 0.779, 95% CI = 0.401–1.158, p < 0.001); (4) control treatment, active treatment (g = 0.511, 95% CI = 0.312–0.710, p < 0.001) and no treatment (g = 0.330, 95% CI = 0.401–1.158, p < 0.001); (5) shift work, night shift (g = 0.439, 95% CI = 0.195–0.684, p < 0.001) and rotating shift (g = 0.430, 95% CI = 0.284–0.576, p < 0.001); (6) simulated shift, simulated (g = 0.454, 95% CI = 0.162–0.746, p = 0.002) and nonsimulated (g = 0.431, 95% CI = 0.290–0.573, p < 0.001); (7) timing of measure, nighttime (g = 0.463, 95% CI = 0.225–0.702, p < 0.001) and day time or mixed (g = 0.410, 95% CI = 0.252–0.569, p < 0.001); (8) research design, randomized controlled trial (g = 0.502, 95% CI = 0.185–0.820, p = 0.002) and randomized crossover trial (g = 0.426, 95% CI = 0.257–0.595, p < 0.001); and (9) data handling, intention-to-treat (g = 0.480, 95% CI = 0.310–0.649, p < 0.001) and per protocol or unknown (g = 0.305, 95% CI = 0.149–0.461, p < 0.001).

For phase shift, the treatment effect was significant in most of the following subgroups (Table 6b): (1) timing of treatment, nighttime (g = 1.528, 95% CI = 0.950–2.107, p < 0.001) and daytime or mixed (g = 0.798, 95% CI = 0.390–1.206, p < 0.001); (2) treatment day, same day (g = 0.967, 95% CI = 0.334–1.599, p = 0.003), different day (g = 1.137, 95% CI = 0.688–1.586, p < 0.001); (3) type of treatment light, single type (g = 1.141, 95% CI = 0.741–1.541, p < 0.001) and mixed type (g = 0.889, 95% CI = 0.062–1.717, p = 0.035); (4) control treatment, active treatment (g = 1.216, 95% CI = 0.848–1.584, p < 0.001), but not significant for no treatment (g = 0.245, 95% CI = − 0.389–0.878, p = 0.449); (5) shift work, night shift (g = 1.164, 95% CI = 0.720–1.607, p < 0.001) and rotating shift (g = 0.892, 95% CI = 0.193–1.592, p = 0.012); (6) simulated shift, simulated (g = 1.379, 95% CI = 1.041–1.718, p < 0.001) and nonsimulated (g = 0.436, 95% CI = 0.071–0.802, p = 0.019); (7) timing of measure, nighttime (g = 1.194, 95% CI = 0.635–1.753, p < 0.001) and daytime or mixed (g = 1.016, 95% CI = 0.592–1.441, p < 0.001); (8) research design, randomized controlled trial (g = 1.447, 95% CI = 0.869–2.025, p < 0.001), randomized crossover trial (g = 0.748, 95% CI = 0.423–1.072, p < 0.001); (9) data handling, intention-to-treat (g = 1.128, 95% CI = 0.398–1.859, p = 0.002) and per protocol or unknown (g = 1.127, 95% CI = 0.827–1.429, p < 0.001). These results suggest that treatment settings and research design significantly influenced treatment effects.

### Moderator analyses of subjective scales

For the subjective scales of sleepiness, higher to lower effect sizes were associated with SSS, VAS, and KSS, respectively (Table 5c): SSS (g = 0.602, 95% CI = 0.354–0.849, p < 0.001), VAS (g = 0.576, 95% CI = 0.190–0.963, p = 0.003), and KSS (g = 0.221, 95% CI = 0.127–0.315, p < 0.001). The findings suggest potential inconsistency in the use of different scales in assessing sleepiness.

## Discussion

The current meta-analysis is the first to investigate the dose–response relationship between light therapy and the circadian phase in shift workers. The results of random-effects models revealed that the pooled effect size of light therapy in reducing sleepiness (15 studies) was small to medium (g = 0.429) and phase shift (13 studies) was large (g = 1.079) in shift workers. In addition, we observed a significant dose–response effect of light therapy on both outcomes, where different intensities and durations of light therapy exerted different effects on sleepiness and phase shift. In particular, light therapy that involved a medium light intensity, shorter duration, and more daily treatment sessions was significantly more effective in reducing sleepiness. These findings suggest a potential saturation of medium light intensity and shorter treatment duration in reducing sleepiness. By contrast, higher-intensity light therapy was significantly more effective in phase shifting; however, whether a longer or shorter treatment duration exerted a better effect on phase shifting remained unclear.

A study on light therapy in healthy participants reported no significant differences in the effects of moderate and high light intensities and longer duration on circadian phase shift^11^. Another study reported that a longer duration of light exposure more effectively reduced sleepiness than shorter durations did^19^. The findings of the current meta-analysis reveal different effects of light therapy on sleepiness and phase shift in shift workers. A moderate intensity and shorter duration of light were more effective in reducing sleepiness, whereas a higher intensity but an unknown duration of light were more effective in sleep phase shifting. The inconsistencies in the results of the current and previous studies may be due to the differences in study populations (inclusion of healthy participants without shift work in previous studies versus those of simulated shift and shift workers in the current study). Furthermore, a higher number of treatment sessions was more effective in reducing sleepiness, whereas a lower number of treatment sessions was more favorable for sleep phase shifting. The current findings are based on the pooled effect sizes of eligible studies on light therapy; thus, they may serve as a reference for establishing the effectiveness of light therapy and its dose–response relationship with sleepiness and circadian phase in shift workers.

Shift workers are usually more prone to sleepiness than nonshift workers are^39^. Light therapy may suppress melatonin secretion and reduce sleepiness in shift workers^11,12^. Moreover, light therapy may shift the circadian phase for sleeping later on workdays and sleeping earlier on days off. In addition, the effects of light therapy on sleepiness varied with the type of shift work. Light therapy was significantly more effective at reducing sleepiness and inducing phase shifting in the night shift than in the rotating shift; this difference may be due to steady changes in the circadian phase experienced by night shift workers. Furthermore, light therapy was more effective at reducing sleepiness and inducing phase shifting in simulated shift workers possibly due to changes initiated in the circadian phase by simulated shifts and because they were more adapted to shift work than nonsimulated shift participants were. Furthermore, light therapy was more effective at reducing sleepiness and inducing phase shifting in simulated shift workers possibly due to changes initiated in the circadian phase by simulated shifts and because they were more adapted to shift work than nonsimulated shift participants were. Therefore, light therapy was more effective at restoring alertness to normal levels and inducing phase shifting in simulated shift workers. Circadian phase shift was characterized by a change in the circadian phase from irregular to relatively normal during the night shift. Moreover, quality assessments indicated that substantially high or unclear risks of methodological biases affected both outcomes in the included studies. Particularly, pooled effect sizes were lower for studies with a high risk of incomplete outcome data. In addition, light therapy exerted better effects on sleepiness and phase shifting in studies that included a randomized controlled design, active control treatment, timing of measure at night, a mixed type of light (for sleepiness) or a single type of light (for phase shifting), treatment at night (for phase shifting) or other times (for sleepiness), and treatment on the same day (for sleepiness) or different day (for phase shifting). Younger people may benefit more in phase shifting after receiving light therapy. These results may serve as a reference for the design and settings of future studies on light therapy.

This meta-analysis had some limitations, and its results must be interpreted with caution. The included studies had high interstudy variation regarding the degree of intensity, duration of treatment, and number of treatment sessions of light therapy; this variation did not provide a sufficient layer of treatment dose of light therapy for a meta-analysis. Currently, we can only report dose–response patterns of a higher or lower degree of treatment doses of light therapy that may be more effective on sleepiness and phase shift in shift workers instead of making conclusive recommendations for the most effective treatment dose. In addition, we examined the effects of different treatment settings and research design on outcomes. These findings may serve as a reference for future experimental studies on the effective treatment dose of light therapy. Regarding data quality, we performed a subgroup analysis to reveal the reasons for potential heterogeneity in pooled effect sizes. Low heterogeneity was observed for most of the significant subgroups of the included studies. Most of the included studies had small sample sizes, and slight variations in studies could change the results. Potential publication bias was found in studies on sleepiness. Therefore, we used random-effects models for adjusting interstudy variations. During data collection, two studies were excluded because of insufficient data, a problem that persisted even after the authors were contacted. Many identified studies were not randomized; therefore, the number of eligible studies in this meta-analysis was limited. In this meta-analysis, we focused on sleepiness and circadian phase shift; however, their intersection could not be examined. For example, the effect of light therapy on changes in sleepiness may be influenced by underlying phase resetting. Moreover, the effect sizes of sleepiness may be influenced by different subjective scales used in the included studies. We performed a subgroup analysis examining this problem, which suggested significant differences among the scales. The outcomes of sleep disorders in shift workers were not investigated. Meta-analyses and experimental studies have insufficiently examined whether light therapy is effective in treating sleep disorders in shift workers.

## Conclusion

The findings of the current meta-analysis suggest that exposure to a moderate intensity and a shorter duration of light therapy was relatively better at reducing sleepiness in shift workers (such as shift nurses) compared with lower- and higher-intensity and longer-duration light therapy, respectively. This finding suggests potential saturation of the treatment dose of light therapy on sleepiness. Exposure to higher-intensity light was more effective at inducing phase shifting compared with low- and moderate-intensity light, but the most effective treatment duration was unclear. Shift workers may mitigate sleepiness and shift sleep phases to achieve better sleep by exposing themselves to bright light at night to improve alertness at work and shift their circadian phase for improved sleep.
